# Uncovering the Role of p38 Family Members in Adipose Tissue Physiology

**DOI:** 10.3389/fendo.2020.572089

**Published:** 2020-12-23

**Authors:** Magdalena Leiva, Nuria Matesanz, Marta Pulgarín-Alfaro, Ivana Nikolic, Guadalupe Sabio

**Affiliations:** Centro Nacional de Investigaciones Cardiovasculares (CNIC), Madrid, Spain

**Keywords:** signaling, kinase, Adipose, Brown, P38, p38 mitogen-activated protein kinase(s)

## Abstract

The complex functions of adipose tissue have been a focus of research interest over the past twenty years. Adipose tissue is not only the main energy storage depot, but also one of the largest endocrine organs in the body and carries out crucial metabolic functions. Moreover, brown and beige adipose depots are major sites of energy expenditure through the activation of adaptive, non-shivering thermogenesis. In recent years, numerous signaling molecules and pathways have emerged as critical regulators of adipose tissue, in both homeostasis and obesity-related disease. Among the best characterized are members of the p38 kinase family. The activity of these kinases has emerged as a key contributor to the biology of the white and brown adipose tissues, and their modulation could provide new therapeutic approaches against obesity. Here, we give an overview of the roles of the distinct p38 family members in adipose tissue, focusing on their actions in adipogenesis, thermogenic activity, and secretory function.

## Introduction

Obesity has become a global pandemic, in part due to lifestyle changes that have brought about an imbalance between energy intake and expenditure. Obesity is characterized by the expansion of adipose tissue through both the hypertrophy of preexisting adipocytes and hyperplasia of adipocyte precursors (1). Adipose tissue has a unique capacity to expand and retract depending on the energy demands (2). This plasticity, unparalleled in other organs, makes adipose tissue the main lipid storage organ. For many years, this was thought to be its only function. However, today adipose tissue is recognized as an indispensable, multi-faceted, and highly metabolically active organ that fulfils a range of functions including mechanical protection and thermal insulation, energy storage, immune responses, endocrine functions, and non-shivering thermogenesis (3). These advances in adipose tissue biology situate the adipocyte as a central rheostat in the regulation of systemic nutrient and energy homeostasis (4).

Stress activated protein kinases (SAPKs) are an important family of mitogen activated protein kinases (MAPKs) that are activated by stress stimuli in a cell-type-dependent manner and transduce stress signals into the cell (5, 6). The SAPKs include the JNK family (JNK1, JNK2, and JNK3) and the p38 family, which numbers four members: p38*α*, p38*β*, p38*γ*, and p38*δ*. SAPKs in adipose tissue of obese individuals have been recognized as important contributors to obesity development and associated insulin resistance. Obesity triggers JNK activation in adipose tissue, where it plays an essential role in adipocyte-mediated insulin resistance (7, 8). In contrast, p38*α* activity is markedly decreased in the adipose tissue of mice with diet-induced or genetically induced (ob/ob) obesity (9). This finding is borne out by low p38*α* expression in human adipose tissue from obese individuals, with the level of this kinase correlating inversely with body mass index (BMI) (10). However, several upstream kinases are upregulated in visceral fat from overweight and obese individuals, including ASK1 and MKK6 (11, 12), suggesting that other p38 isoforms might be activated in these conditions.

p38 pathway is one of the main proposed controllers of the activation of brown adipose tissue (BAT) and the browning of white adipose tissue (WAT). In both processes, p38 signaling operates by inducing uncoupling protein 1 (UCP1) transcription through the activation of cAMP response element-binding protein (CREB), activating transcription factor 2 (ATF2), and peroxisome proliferator-activated receptor gamma coactivator 1α (PGC1*α*) (13). WAT browning and BAT activation are thought to require activation of the p38 cascade by *β*-adrenergic stimuli or other browning agents (13–22). However, it remains unclear what roles are played in these processes by each of the p38 family members, which have distinct physiological functions and expression patterns. It is therefore vital to determine the precise regulation of this pathway and to identify which p38 and upstream kinases are implicated in WAT browning and BAT activation. Recent results obtained in our laboratory indicate that the activation of each p38 is tissue- and upstream–kinase-dependent. For example, all p38s in WAT are activated mostly by MKK3, and mice lacking this kinase show a robust decrease in p38 activation; however, we recently showed that in BAT, p38 activation is mainly dependent on MKK6 (12). Interestingly, in obesity, MKK6 expression increases in WAT but decreases in BAT. These data suggest that p38 regulation differs markedly between BAT and WAT.

## The p38 Mapk Pathway and Adipogenesis

Knowledge about the origin of white and brown adipocytes has been substantially revised in the last few years. Although brown and white adipocytes share a mesodermal origin, they originate from different populations of embryonic multipotent mesenchymal stem cells (MSCs) (23, 24). Due to its function in thermogenesis, especially in newborns, BAT develops before birth, and brown adipocytes originate from the myogenic Myf5-positive MSC lineage (25). White preadipocytes arise from adipogenic Myf5-negative MSCs and differentiate to WAT shortly after birth (26, 27). Despite this difference in MSC origin, adipogenesis in both white and brown adipocytes involves the activation of the same key transcription factors: peroxisome proliferator-activated receptor *γ* (PPAR*γ*) and CCAAT/enhancer-binding proteins (C/EBP) (28, 29). Adipocyte differentiation is also regulated by bone morphogenetic proteins (BMP), members of the transforming growth factor (TGF)-*β* superfamily. Differentiation of white adipocytes is regulated by BMPs 2 and 4 (30–32), whereas BMP7 and PR domain containing 16 (PRDM16) are crucial factors in the differentiation of preadipocytes to mature brown adipocytes (17, 33). Another common regulator of brown and white adipocyte differentiation is the p38 pathway, and here we will summarize the current knowledge about the role of this kinase family in WAT and BAT adipogenesis.

## p38 Protein Kinase Family Controls White Adipogenesis

The precise role of p38 kinases in adipogenesis remains unclear, with some studies indicating that p38 activation promotes adipogenesis (34–36) while others suggesting that it inhibits this process (9, 37, 38). More than two decades ago, pioneering work by Engelman and colleagues demonstrated that p38 orchestrates the early steps in the differentiation of 3T3-L1 fibroblasts to adipocytes by activating the transcription factor C/EBP*β*. These authors found that pharmacological inhibition of p38 blocked the initial stages of adipogenesis but did not affect the later stages of differentiation (34). Work by the same group also demonstrated the important contribution of p38 to adipogenesis and the consequent need to tightly regulate its activity during this process. This work showed that p38 activation, by salicylate or active MKK6, spontaneously triggers adipogenesis of 3T3-L1 cells and that uncontrolled and prolonged activation of p38 results in massive cell death (36). Nevertheless, other studies have demonstrated a negative role of p38 in adipogenesis (39). p38 phosphorylates the C/EBP homologous protein (CHOP), a negative regulator of C/EBP*α* (38), and nuclear factor of activated T cells 4 (NFATc4) (37), both of which regulate adipocyte differentiation. In addition, Aouadi and colleagues found that p38 activity declines during adipocyte differentiation and that pharmacological or genetic p38*α* deficiency stimulates adipogenesis *in vitro* and *in vivo*. The anti-adipogenic effects of p38 are due to inhibition of C/EBP*β* and PPAR*γ* activity (9). Contrasting the mouse results, the same authors demonstrated a positive role of p38 in human preadipocyte differentiation, showing that pharmacological inhibition of p38 reduced C/EBP*β* phosphorylation, PPAR*γ* expression, and lipid accumulation in primary human preadipocytes (35).

To evaluate the alteration of nuclear proteins during early stages of 3T3-L1 preadipocyte differentiation, Rabiee et al. performed a proteomic and phosphoproteomic analysis. After generating a kinase-substrate database, these authors showed that most putative protein kinases involved in early adipogenesis belong to the cyclin-dependent kinase (CDK) family and the MAPK family, which includes p38 and JNK (40). Further analysis showed that most transcriptional regulators are phosphorylated during early adipogenesis and that most of the phosphorylated peptides included the consensus motif S(p)/T(p)-P. These are target sequences for CDKs and MAPKs, thus establishing the fundamental role of MAPKs in adipogenesis. Interestingly, we recently demonstrated that p38*γ* and p38*δ* can act as CDKs and that the CDK and p38 kinase families cooperate in the phosphorylation of their substrates in the liver (41). It would be interesting to assess whether these kinase families also jointly coordinate the differentiation of adipose tissue.

**Figure 1:**
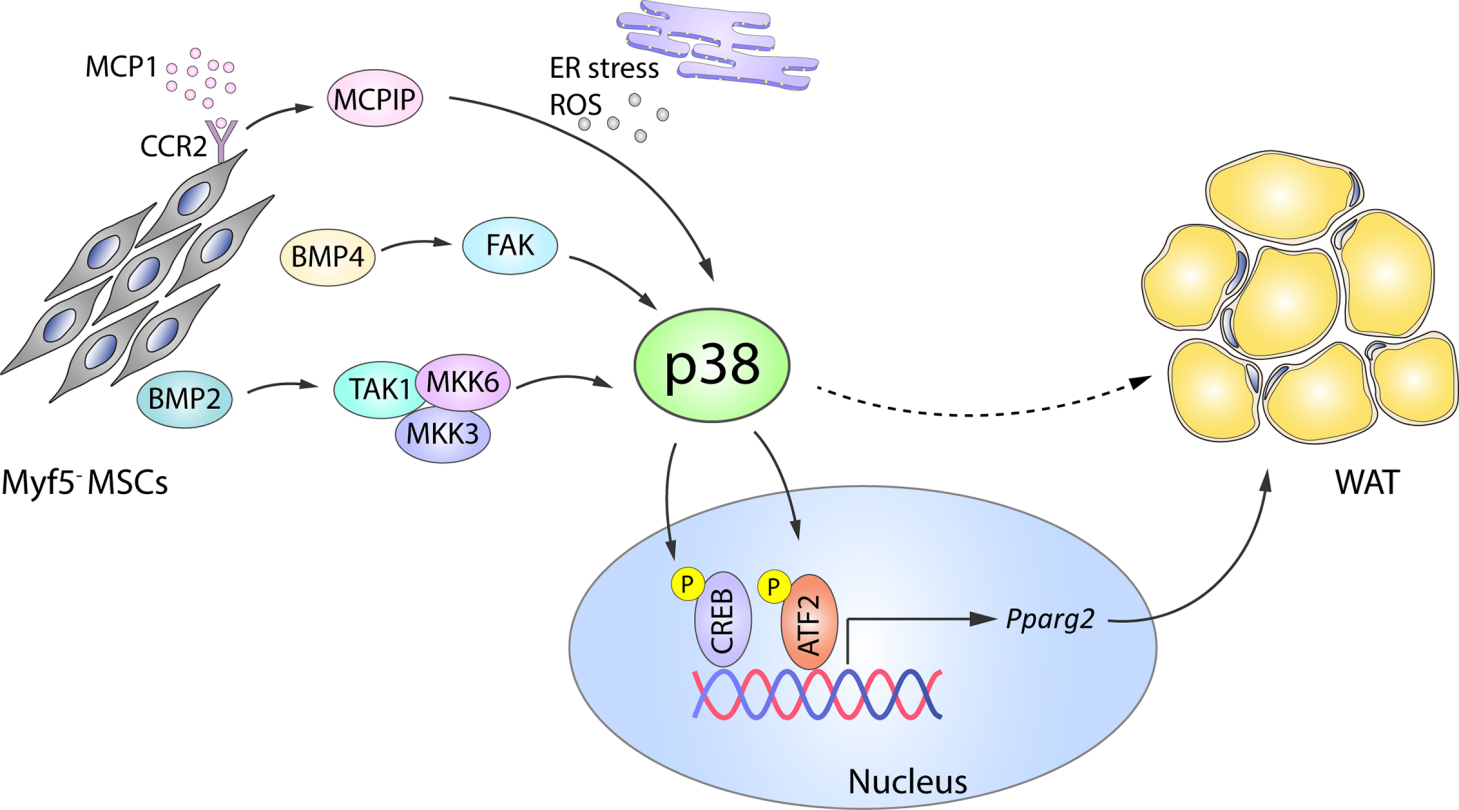
Role of p38 in WAT adipogenesis. In Myf5^-^ MSC cells, p38 protein kinase family members are activated by a variety of upstream activators, including BMP2–TAK1–MKK3/6, BMP4–FAK, and MCP1–MCPIP. p38-mediated phosphorylation and activation of CREB and ATF2 then leads to increased expression of *Pparg2* and WAT adipogenesis. Solid arrows represent the direct effects of molecular players involved in the indicated signaling pathways, while dotted arrows represent indirect effects, meaning that other unknown molecules might be involved. ATF2, activating transcription factor 2;BMP, bone morphogenetic proteins; CREB, cAMP response element-binding; ER, endoplasmic reticulum; FAK, focal adhesion kinase; MCP1, monocyte chemotactic protein 1; MCPIP, MCP1-induced protein; MKK, mitogen-activated protein kinase kinase; ROS, reactive oxygen species; MSC, mesenchymal stem cell; *Pparg2*, peroxisome proliferator-activated receptor gamma 2; TAK1, transforming growth factor beta-activated kinase 1; WAT, white adipose tissue. Yellow circled ‘P’ indicates phosphorylation.

The transcription factor BMP4 plays an important role in white adipocyte development by triggering MSC commitment to adipocyte fate (42, 43), inhibiting the brown phenotype during terminal differentiation, and promoting a shift from the brown adipocyte phenotype towards a white-like phenotype (32). BMP4 and the related BMP2 mediate adipogenic function *via* two main signaling pathways: the canonical SMAD pathway (44) and a SMAD-independent pathway that involves activation of p38 by the upstream activators transforming growth factor beta-activated kinase 1 (TAK1), MKK3, and MKK6 (45). BMP2-induced p38 activation (45) leads to the phosphorylation of its downstream substrate ATF2, which in turn regulates PPAR*γ*2 expression and adipogenesis (**Figure 1**). In consequence, ATF2 deletion or the chemical inhibition of p38*α*/*β* in mice results in decreased PPAR*γ*2 expression and adipocyte differentiation *in vitro*. The reduced WAT content in these mice protects them against high fat diet (HFD)-induced obesity (46). In addition to these mechanisms, BMP4-induced activation of SMAD and p38s was recently shown to induce focal adhesion kinase (FAK) in early adipogenesis, and FAK silencing or inhibition downregulated adipogenesis while also reducing SMAD and p38 activation (47). These findings suggest that there are more molecular players in these complex processes yet to be discovered (**Figure 1**).

p38 signaling is also implicated in the adipogenic action of monocyte chemotactic protein 1 (MCP1)-induced protein (MCPIC). MCP1 and its receptor CCR2 are produced in preadipocytes during *in vitro* adipogenesis and contribute to adipogenesis through the induction of MCPIP (48). During the later stages of adipogenesis, MCPIP triggers the endoplasmic reticulum stress response, autophagy, ROS induction, and p38 activation. Chemical inhibition of p38 in 3T3-L1 cells attenuates the expression of adipogenic markers (adiponectin and lipoprotein lipase) in MCPIP-induced adipogenesis (49), suggesting a pro-adipogenic role of the p38 family (**Figure 1**).

## p38 SAPKs As Regulators of Brown Adipogenesis

**Figure 2:**
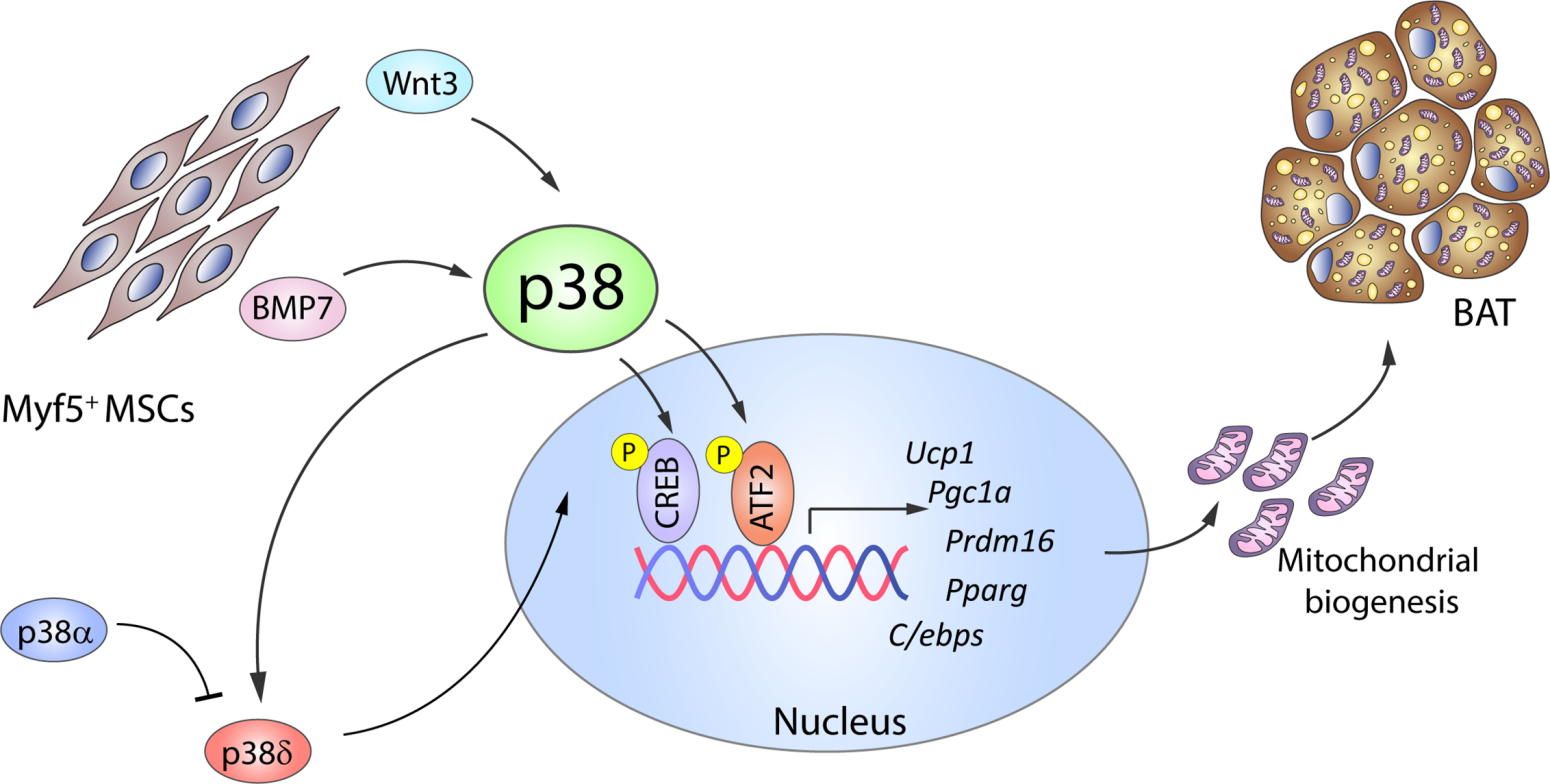
The p38 kinase family controls BAT adipogenesis. Brown adipocytes originate from Myf5^+^ MSC cells. BMP7 and Wnt3 activate p38 protein kinases, which phosphorylate and activate CREB and ATF2, leading to increased gene expression of brown adipocyte signature markers and mitochondrial biogenesis. p38*δ* has been suggested as the main kinase promoting BAT activation, whereas p38*α* has opposing effects. Black arrows represent the direct effects of molecular players involved in the indicated signaling pathways or effects. ATF2, activating transcription factor 2; BAT, brown adipose tissue; BMP, bone morphogenetic protein; *C/ebp*, CCAAT/enhancer-binding protein; CREB, cAMP response element-binding; MSC, mesenchymal stem cell; *Ppargc1a*, peroxisome proliferator-activated receptor gamma coactivator 1*α*; *Prdm16*, PR domain containing 16; *Pparg*, peroxisome proliferator-activated receptor; *Ucp1*, uncoupling protein 1. Yellow circled ‘P’ indicates phosphorylation.

Significant progress has also been made in understanding the molecular mechanisms of brown adipogenesis. BMP2 and BMP4 have been reported to promote white adipogenesis in MSCs or preadipocytes *in vitro* when exposed to an adipogenic cocktail (50, 51); however, Tseng et al. showed that in the absence of the adipogenic cocktail, treatment with BMP2, BMP4, BMP6, or BMP7 promotes lipid accumulation in brown but not white preadipocytes (17). Of these BMPs, BMP7 is the only one that substantially increases the expression of UCP1, other early brown-fat fate regulators like PRDM16 and PGC1*α*, and the adipogenic transcription factors PPARγ and C/EBPs, as well as inducing mitochondrial biogenesis (17). The same study also established that BMP7-induced brown adipogenesis requires p38 and PGC1*α*: BMP7 treatment of brown preadipocytes activated p38, leading to phosphorylation of its substrate ATF2 (**Figure 2**), whereas p38 inhibition reduced BMP-7-induced UCP1 expression (17). A recent comparative epigenome and transcriptome profiling of C3H10T1/2 mesenchymal cells during differentiation into thermogenic brown adipocytes revealed that BMP7–p38 signaling potentially targets Sox genes, which are important for early lineage commitment of multipotent progenitors to brown adipocytes (52). This study confirmed that Sox13 promotes adipogenic differentiation, brown marker gene expression, and mitochondrial respiration (52).

p38–CREB-mediated mitochondrial biogenesis is also promoted by the adipogenesis regulator Wnt3a. Treatment of *in vitro* differentiated 3T3-L1 adipocytes with Wnt3a upregulates the expression of mitochondrial genes and mitochondrial copy number in a process independent of the canonical Wnt/*β*-catenin pathway (53). Instead, Wnt3a induces rapid and transient activation of p38 (54, 55), and pharmacological inhibition of p38 reduces CREB activation and attenuates the observed effects of Wnt3a (**Figure 2**).

A number of naturally occurring compounds with pro- or anti-adipogenic effects on white and brown adipogenesis have been found to act mainly through the AMPK*α* and p38 pathways. These compounds include phloretin (56), sinigrin (57), and cryptotanshione (58). Phloretin is a glucose transporter inhibitor found in some fruits that enhances adipogenesis in the bone-marrow derived stromal cell line ST2 by inhibiting ERK and JNK and activating p38 (56). Singirin, a glucoside found in broccoli, Brussels sprouts and black mustard seeds, has the opposite effect, inhibiting the early stage adipogenesis of 3T3-L1 adipocytes by activating AMPK, MAPKs, and acetyl-CoA carboxylase (57). Cryptotanshione, found in *Salvia miltiorrhiza*, stimulates brown adipogenesis and inhibits WAT-specific markers through AMPK*α*, p38*α*, and SMAD signaling (58).

## Adipocyte Plasticity and Transdifferentiation

Adipocyte-like cells can also be generated by the transdifferentiation of muscle satellite cells (59, 60), and this complex reprograming process is reversible (61). p38 kinases are candidate negative regulators of the broad molecular network involved. The p38 pathway is activated in early and late stages of transdifferentiation, and pharmacological inhibition of p38*α*/*β* by SB203580 stimulates adipogenic metabolism, increases lipid production, and promotes expression of adipogenic regulators (62). In addition, dominant-negative MKK3 stimulates transdifferentiation of C2C12 myogenic cells into adipocytes *via* upregulation of PPAR*γ* and the phosphoinositide-3 kinase pathway (59). Furthermore, during pregnancy and lactation subcutaneous white adipocytes can also transdifferentiate into mammary glands and so-called pink adipocytes, responsible for milk production (63, 64) and this process is reversible after lactation (65). As discussed in this review, p38 protein kinase family has an important role in adipose tissue plasticity, and it would be interesting to examine the role of p38s in development and function of pink adipocytes.

Until recently, most evidence implicating p38 kinases in adipogenesis came from experiments with pharmacological inhibitors. However, kinase inhibitors have important limitations due to their lack of specificity and possible side effects. Using adipose p38*α* knock out mice, we recently showed that lack of p38*α* results in reduced weight of several fat depots, suggesting a possible positive role of p38*α* in adipogenesis *in vivo* (10). These results were subsequently confirmed by another study (66). We also demonstrated that deletion of p38*α* in adipose tissue leads to a reduced content of BAT and several types of WAT, whereas the lack of p38*δ* has opposing effects (10). Moreover, *in vitro*-differentiated brown preadipocytes lacking p38*α* have higher expression of brown adipocyte markers and mitochondrial genes, whereas lack of p38*δ* downregulates BAT signature genes in differentiated brown adipocytes (10). These data highlight a novel role of p38*δ* as a positive inducer of brown adipogenesis. The opposite roles of p38*α* and p38*δ* in brown adipogenesis underline the need for further research using genetically modified mice to define the actions of individual p38 family members in adipogenesis, both in early and late stages.

## p38 in BAT Activation

BAT is an important organ involved in fat burning and body-temperature maintenance through non-shivering thermogenesis (67). Although the presence of BAT in small mammals and human newborns was well established, it was believed to lose its function in adult humans. A decade ago, positron emission computed tomography studies revealed certain areas of the adult human body with a very active uptake of fluorodeoxy-glucose (68). These regions were identified as BAT depots, and are mainly located in cervical, supraclavicular, axillary, and paravertebral regions (69). The amount and the activity of BAT are dependent on several factors, such as age, leanness, and environmental temperature, and obese individuals have deficiencies in BAT activation and browning (68).

Mice with increased BAT activity are resistant to metabolic diseases, not only because they expend more energy on thermogenesis, but also because of improvements in systemic metabolism resulting from BAT actively removing glucose and lipids from the bloodstream and thereby increasing glucose tolerance and insulin sensitivity (69). This evidence suggests that modulation of BAT activity offers a possible therapeutic strategy for increasing energy expenditure and to achieving a negative energy balance, as well as an improved metabolic status.

## p38 Signaling During BAT Activation

Whereas white adipocytes store lipids in a single large triglyceride-filled droplet and contain few mitochondria, brown adipocytes store lipids in multiple small droplets and contain a large number of mitochondria (67). In BAT, thermogenesis is mainly controlled by UCP1, a mitochondrial protein that uncouples the respiratory chain from ATP synthesis, so that the energy generated is dissipated as heat (13). BAT is activated by a variety of stimuli, including cold, hormones, and certain food components, all of which trigger an increase in proton flux through UCP1, resulting in heat production (70). BAT activation triggers a cell signal transduction that causes not only acute effects such as enhanced lipolysis and UCP1 activity, but also chronic effects that include increased expression of thermogenic genes, mitochondrial biogenesis, and neoplastic and hyperplasic growth of brown adipocytes (67, 71).

**Figure 3:**
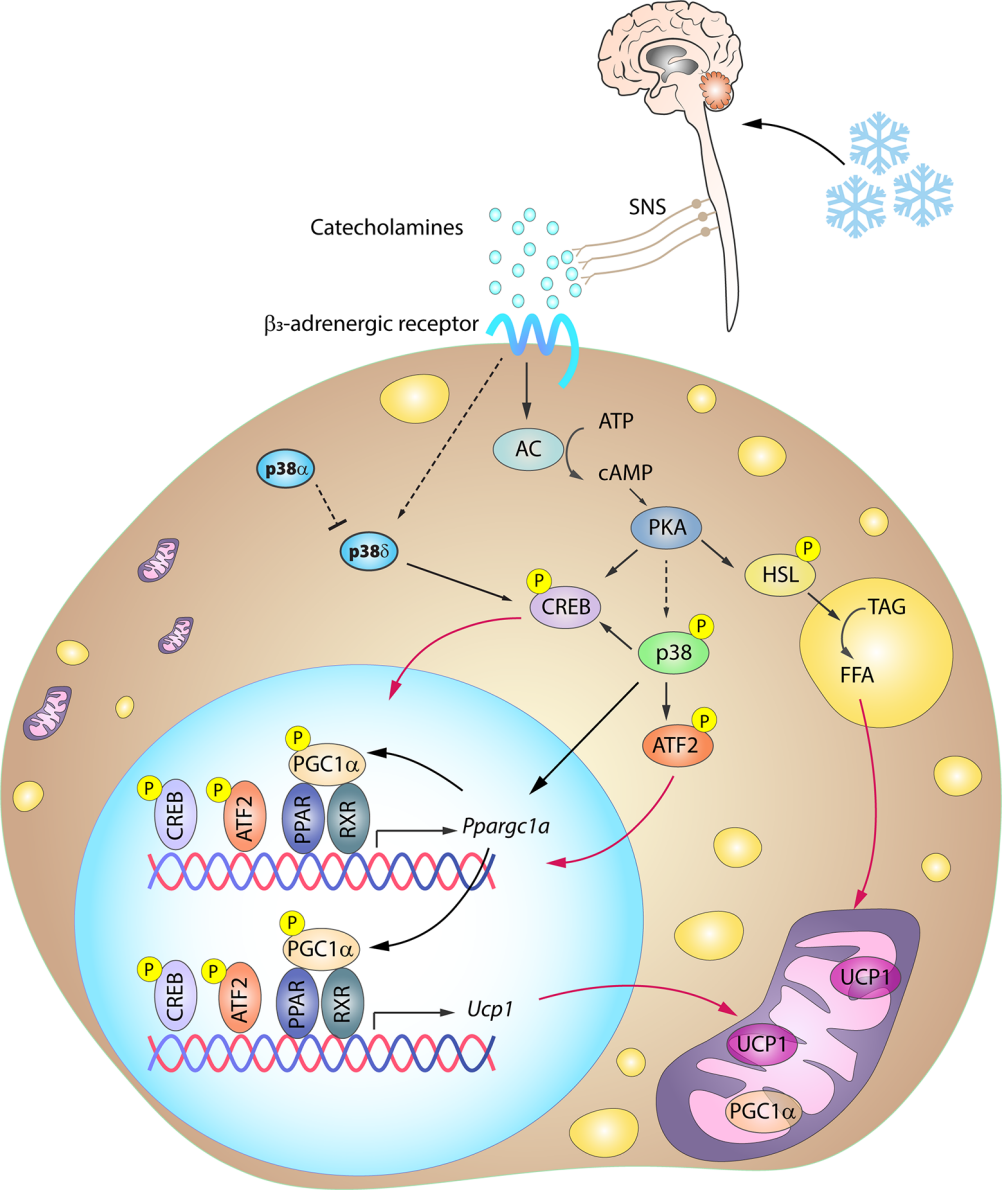
Simplified scheme of the signaling cascade for BAT activation in response to cold. Cold-induced release of catecholamines by the SNS activates thermogenesis in brown adipocytes by stimulating *β*3-adrenergic receptors, which trigger PKA activation through an increase in intracellular cAMP. PKA participates in the activation of several transcription factors involved in the BAT thermogenic response. p38-mediated phosphorylation of several of these transcription factors is necessary for the expression of BAT signature genes. Solid arrows represent the direct effects of molecular players involved in the indicated signaling pathways, dotted arrows represent indirect effects, meaning that other unknown molecules might be involved, while red arrows represent translocation between cell compartments. AC, adenylyl cyclase; ATF2, activating transcription factor 2; cAMP, cyclic AMP; CREB, cAMP response element-binding; HSL, hormone sensitive lipase; PGC1*α*/*Ppargc1a*, peroxisome proliferator-activated receptor gamma coactivator 1α; PKA, protein kinase A; PPAR, peroxisome proliferator-activated receptor; RXR, retinoid X receptor; SNS, sympathetic nervous system; TAG, triglycerides; UCP1, uncoupling protein 1. Yellow circled ‘P’ indicates phosphorylation.

One of the key signaling proteins involved in BAT activation is protein kinase A (PKA). Activated PKA phosphorylates hormone sensitive lipase (HSL), stimulating its activity and in turn promoting lipolysis. The resulting free fatty acids (FFA) undergo *β*-oxidation, and the increase in FFA levels also promotes UCP1 activity (72–74). Additionally, PKA participates in the activation of the p38 pathway (15), which contributes to BAT thermogenesis through the phosphorylation of key transcription factors driving UCP1 expression (12–14, 17, 18). Three well-recognized transcription factors within this group are PGC1*α*, ATF2 and CREB. PGC1*α* is an important regulator of mitochondrial biogenesis and oxidative phosphorylation (75, 76) and is also required for enhanced UCP1 expression. p38 promotes PGC1*α* transcription by phosphorylating ATF2 and CREB, which bind as homodimers to cAMP response elements (CREs) in the PGC1*α* promoter (13, 77). CREB activation was thought to be p38 independent because PKA can promote its activation by direct phosphorylation at Ser133 (13, 15). However, CREB can be also an indirect target of p38 through activation of the p38 substrate MSK1 (78). Moreover, p38 also directly phosphorylates PGC1*α*, enhancing its stability and activity (79). Phosphorylated PGC1*α* acts as a co-activator by interacting with different dimer combinations of PPAR*α*, PPAR*γ*, retinoid X receptor, retinoic acid receptor, and thyroid receptor transcription factors (19, 71, 80). These complexes bind to PPAR response elements present in both the PGC1*α* and the UCP1 promoter, and PGC1*α* thus acts as a co-activator not only for its own transcription in an autoregulatory loop, but also for UCP1 transcription (81). p38-mediated phosphorylation of ATF2 and CREB also allows these transcription factors to bind to CRE sites in the UCP1 promoter, increasing UCP1 expression (13). In light of this accumulated evidence, it seems undeniable that the p38 pathway plays a central role in the coordinated thermogenic response (**Figure 3**).

Recent research has identified another mechanism of p38-mediated UCP1 regulation. p38-mediated phosphorylation of the transcription factor Zc3h10 at S126 induces its binding to the UCP1 promoter at a ~4.6 kb upstream region, increasing UCP1 expression and thermogenesis (82).

## Signaling Triggering BAT Thermogenesis Through p38

Cold-induced BAT activation and thermogenesis mainly relies on sympathetic nervous system (SNS) innervation. Upon cold exposure, catecholamines released by the SNS activate the thermogenic program in brown adipocytes by stimulating *β*_3_-adrenergic receptors (*β*3AR) (68), which are abundantly expressed in this cell type. *β*3AR stimulation is followed by an adenylate cyclase (AC)-mediated increase in intracellular cAMP. The resulting activation of PKA is associated with increased expression of thermogenic genes *via* p38 activation (**Figure 3**).

BAT activity is also promoted by other factors, secreted either BAT or other organs. Thyroid hormones (TH) activate BAT by both TH-mediated SNS stimulation and peripheral actions (83). In brown adipocytes, T4 is converted by 5-deiodinase type 2 (Dio2) to T3, which binds to TH receptors located in TH response elements (TREs) present in the promoters of thermogenic genes such as UCP1 (84). T3 thus acts synergistically with *β*3-agonists to induce BAT activity. T3 also promotes fatty acid-oxidation, lipogenesis, and mitochondrial biogenesis in BAT (83), enhancing its thermogenic capacity. Although the main effects of T3 on BAT activation do not seem to rely on p38 signaling, our group demonstrated that p38 pathway mediates T3-induced browning in WAT (see below) (12).

In addition to their role in BAT differentiation, BMPs also regulate BAT activation. The canonical BMP signaling pathway depends on BMP binding to type I and II BMP receptors, which triggers activation of the SMAD pathway. However, non-canonical BMP signaling also activates the ERK, JNK, and p38 pathways (85). BMP8B is particularly important in the context of BAT activation. Whittle et al. demonstrated that BMP8B expression is upregulated in BAT after cold exposure or HFD, enhancing the response to adrenergic stimulation by increasing p38–CREB signaling and HSL activity. In that study, the authors showed that *Bmp8b*^−/−^ mice exhibit impaired thermogenesis and have a higher susceptibility to diet-induced obesity, together with decreased activation of p38–CREB signaling. However, phosphorylation of the p38 upstream kinases MKK3 and MKK6 is increased, suggesting that BMP8B deletion leads to a blockade at this point in the signaling cascade (18).

A parallel pathway that seems to operate in synergy with β_3_-adrenergic activation is mediated by natriuretic peptides (NPs). NPs are hormones secreted by the heart that bind to NP receptor A (NPRA), leading to an increase in cGMP and subsequent PKG activation, which in turn enhances UCP1 expression in BAT and other mitochondrial markers *via* p38 (86). However, the vast majority of studies related to the role of NPs in thermogenesis have been carried out in the context of browning.

Since BAT activation has emerged as a potential therapeutic approach for the treatment of obesity and other related diseases, immense efforts are currently directed towards finding new compounds able to activate BAT thermogenesis and browning. A number of compounds have been already identified, including menthol, capsaicin, and resveratrol (87–89). Ravaud et al. showed that the p38 pathway is required for resveratrol-induced UCP1 expression, whereas the HIV-protease inhibitor lopinavir has an opposing action associated with reduced p38 phosphorylation (90). Sinapic acid, a natural alkaloidal amine found in black mustard seeds, wine, and vinegar, was recently shown to promote thermogenesis and lipolysis in BAT *via* PKA–p38 signaling (91). However, further studies are needed to provide greater insight into the potential involvement of the p38 pathway in the induction of BAT thermogenesis *via* these compounds.

## Function of p38s in BAT Thermogenesis

Despite the many studies exploring the role of p38 pathway in BAT thermogenesis, few of them have assessed the specific roles of the individual p38 family members in BAT activation. In 2005, Robidoux and colleagues (92) reported that p38*α* is the main p38 responsible for BAT activation. However, this study was based on *in vitro* approaches and *in vivo* experiments with small interfering RNAs (siRNA) and non-specific chemical compounds that inhibit not only p38*α*, but also p38*β*. Analysis by our group and others using genetically modified mouse models demonstrated that p38*α* deletion results in the upregulation of BAT signature genes in brown adipocytes in a cell-autonomous manner (10, 66). We also found that lack of p38*α* induces p38*δ* activation, resulting in increased BAT thermogenesis and energy expenditure. Thus, p38*δ*, and not p38*α*, seems to be the main p38 kinase implicated in the induction of BAT thermogenesis in mouse models (**Figure 3**).

## p38 and White Adipose Tissue Browning

In addition to the established classification of fat depots as WAT and BAT, a third type of fat cell—called brite, beige, or brown-like adipocytes—has been described in recent years (19, 93). Beige adipocytes are characterized by multilocular lipid droplets, high mitochondrial content and metabolic rate, elevated UCP1 expression, and a thermogenic ability similar to BAT. Beige adipocytes arise in WAT depots through a process called browning (70, 94), and the induction of WAT browning is a promising therapeutic strategy for increasing total energy expenditure in metabolic disease (95, 96), in which the capacity for browning is reduced (97, 98). Browning was first described by Young et al. (99) in cold-exposed parametrial fat pads. Since then, the presence of beige adipocytes has been reported in other white fat depots, including epididymal and peri-renal fat (100–102) and especially subcutaneous fat, the fat depot most susceptible to generating brown-like cells in mice (19, 103). In adult humans, most BAT is in fact composed of beige adipocytes (94, 104), and a recent report found browning markers to be more highly expressed in visceral than in subcutaneous WAT, opposite to the situation in mice, suggesting that visceral adipose tissue is the brownest depot in humans (105). Since visceral fat is the main fat linked to metabolic diseases (106), these results enhance the importance of WAT browning as a potential therapeutic strategy. Interestingly, WAT browning seems to differ between the sexes and to change with the seasons (107–110).

It is still unclear what specific mechanisms drive browning: differentiation of pre-existing brown adipocyte precursors within WAT, differentiation from white adipocyte precursors, or transdifferentiation from mature white adipocytes (111–114). A non-BAT origin is supported by the fact that BAT arises from Myf5-positive progenitors, whereas the progenitors of beige adipocytes are Myf5-negative (19). In addition to brown signature markers, beige adipocytes also express beige-specific markers, thus defining a beige fat phenotype different from BAT (94, 111, 115, 116).

**Figure 4:**
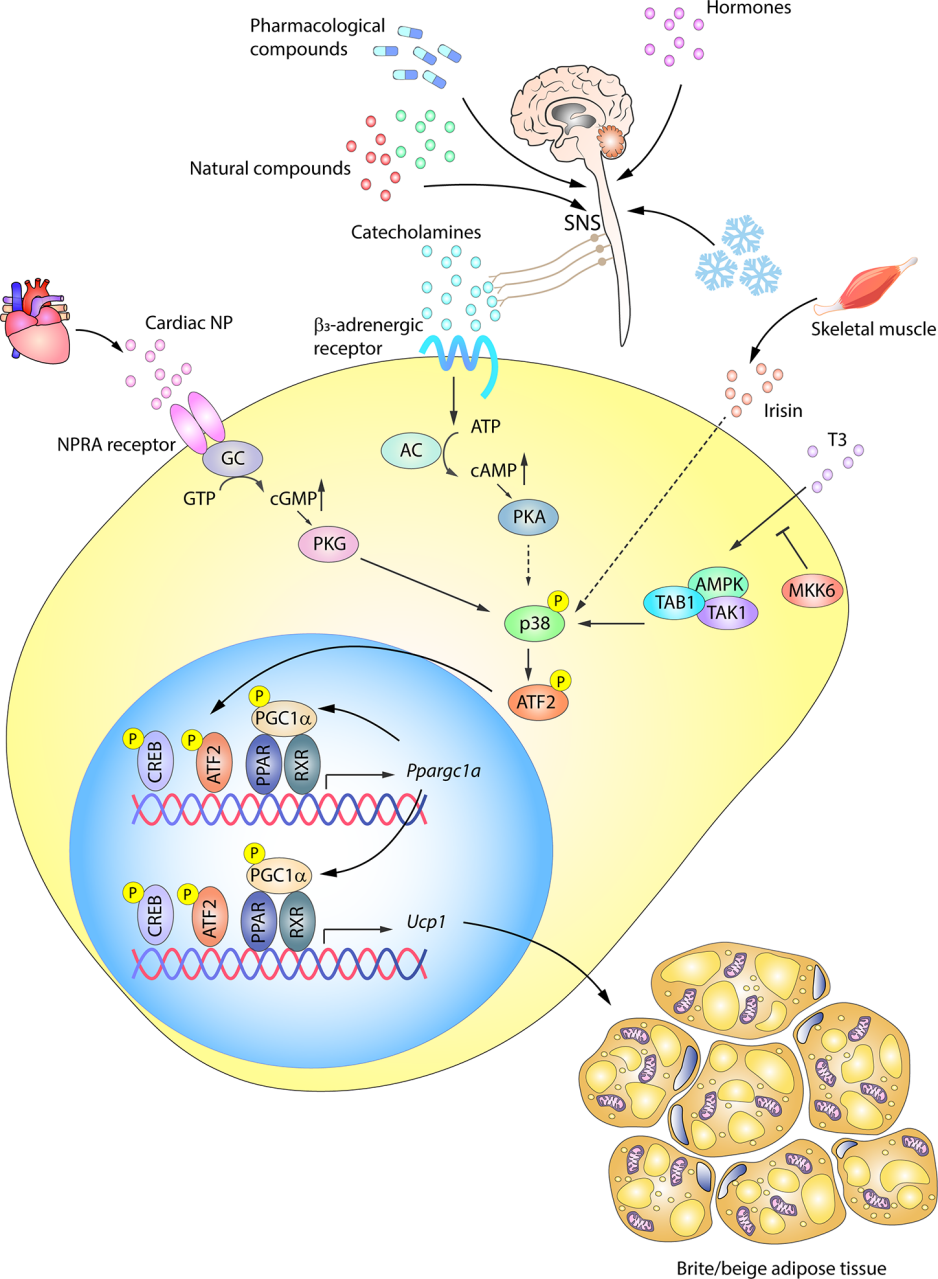
p38-signaling in white adipose tissue browning. WAT browning can be triggered by a variety of stimuli that converge on p38 signaling. In response to cold, hormones, drugs, or naturally occurring compounds, the SNS releases catecholamines to induce WAT browning by stimulating *β*3-adrenergic receptors. The ensuing PKA activation in turn activates p38. p38 phosphorylation and activation can alternatively be triggered by other molecules, such as cardiac NP acting *via* PKG, T3 hormone *via* an alternative pathway, or irisin released by skeletal muscle. Solid arrows represent the direct effects of molecular players involved in the indicated signaling pathways, while dotted arrows represent indirect effects, meaning that other unknown molecules might be involved. Active p38 phosphorylates the transcription factor ATF2, which translocates to the nucleus and upregulates the transcription of UCP1 and other key genes essential for transforming white adipocytes into beige adipocytes. AC, adenylate cyclase; AMPK, AMP-activated protein kinase; ATF2, activating transcription factor 2; cAMP, cyclic AMP; GC, guanylate cyclase; cGMP, cyclic GMP; CREB, cAMP response element-binding; MKK6, mitogen-activated protein kinase kinase 6; NP, natriuretic peptides; PGC1*α*/*Ppargc1a*, peroxisome proliferator-activated receptor gamma coactivator 1α; PKA, protein kinase A; PKG, protein kinase G; PPAR, peroxisome proliferator-activated receptor; RXR, retinoid X receptor; SNS, sympathetic nervous system; TAB1, TAK1 binding protein 1; TAK1, transforming growth factor beta-activated kinase 1; *Ucp1*, uncoupling protein 1. Yellow circled ‘P’ indicates phosphorylation.

WAT browning is an adaptive mechanism activated by a range of stress situations. Aside from cold, other stimuli include exercise, hormones, pharmacological activation, and numerous natural compounds (1, 117–121). All these stimuli, despite activating browning *via* distinct internal mediators, converge on the expression of UCP1, mitochondrial biogenesis, and FFA oxidation, similar to the transformation occurring in BAT activation (74, 117). p38 signaling has been shown to be an important pathway controlling UCP1 expression in browning (**Figure 4**).

*β*3AR activation in WAT was thought to be primarily related to lipolysis, activating HSL and inhibiting perilipin to provide FFA during fasting (122). However, recent work has shown that WAT, like BAT, is highly innervated by the SNS and that adrenergic stimulation (by cold, nutrients, or drugs) is also involved in WAT browning in mice (70, 94, 123–127) and humans (128). The SNS induces the release of cathecolamines like norepinephrine, which, through binding to *β*3AR, trigger PKA and p38 activation and promote UCP1 transcription partly *via* p38-mediated modulation of ATF2 and PGC1*α* transcriptional activity (129) (**Figure 4**). UCP1 expression is also activated *via β*3AR-stimulated p38 activation by a series of naturally occurring compounds, such as thymol (130), cinnamaldehyde (131), L-rhamnose (132), grape pomace extract (133), and mangiferin (134). Along with *β*3AR pathway induction, cold-induced WAT browning is also mediated by stimulation of the histamine H4 receptor, which acts through intracellular calcium mobilization and p38 and ERK activation, promoting PGC1*α* and UCP1 expression (135).

Besides, the *β*3AR–PKA–p38 pathway, a parallel browning system also converging on p38 was described by Bordicchia et al. (86). Cardiac NPs, known as controllers of hemodynamic homeostasis (136), are increased by cold stimulation and activate NPRA, which triggers cGMP synthesis and subsequent PKG activation, leading to p38 phosphorylation and activation of the WAT thermogenic program *via* ATF2 (86, 136). Mice with high NP signaling in adipose tissue are thus protected against diet-induced obesity and insulin resistance (137), revealing an important heart–adipose connection in the regulation of energy metabolism (136). In this way, catecholamines and NPs are able to act synergistically to promote WAT browning, not only through p38, but also through mTORC1 in an Akt-independent manner (138, 139).

WAT browning can also be activated by TH, either indirectly through enhanced adrenergic induction of UCP1 (140–142) or by direct action on white adipocytes (12, 143, 144). Recent work by our group revealed a new regulation of T3 activity-induced browning in white adipocytes in which p38 plays a prominent role (12). Mice lacking the p38-upstream activator MKK6 are resistant to obesity and show elevated browning; these effects are reversed by propylthiouracil (PTU)-mediated inhibition of TH production and restored by treatment with exogenous T3, showing that the phenotype is due to increased T3 sensitivity. Furthermore, *Mkk6* deletion increases T3-stimulated UCP1 expression in white adipocytes through the activation of p38 *via* an alternative pathway involving AMPK, TAK, and TAK1 binding protein 1 (TAB1). In obese patients, MKK6 expression is increased and blocks T3-mediated UCP1 induction, preventing WAT browning in the overfed state (12).

WAT browning in mice is also linked to exercise, although human studies have failed to show an effect after endurance training (145). Browning involves several endocrine mediators released from skeletal muscle, the most notable being irisin, despite controversy about its physiological relevance in humans (146–148). Increased irisin levels in exercised mice (12) enhance total body energy expenditure, activate the thermogenic program *in vitro*, increase lipolysis, and induce a browning phenotype in obesity (80, 149, 150). Irisin-stimulated browning in mice was reported to require p38 signaling (80, 151) (**Figure 4**). In addition, carnosine, a dipeptide abundant in muscle, brain and other tissues, might induce browning and thermogenesis in obese rats through the stimulation of irisin, which up-regulates UCP1, PGC1*α*, and CD137 *via* p38 in inguinal WAT (152).

WAT browning is also promoted by BMPs. BMP7 induces UCP1 in mouse subcutaneous WAT and in human adipocytes (153, 154), hinting that the BMP7–p38 signaling mechanism operating during BAT adipogenesis might also operate in WAT (17). BMP4 is expressed in human subcutaneous and visceral fat, and its expression correlates inversely with BMI (155), suggesting that hypertrophic obesity is a condition of pre-adipocyte resistance to BMP4 (156). BMP4 stimulates browning in mouse and human white pre-adipocytes, and its overexpression in mice increases inguinal WAT browning (153, 155, 157). This induction was reported to be mediated by p38–ATF2 signaling and activation of PGC1*α* (155). Growth differentiation factor 5 (GDF5), another TGF-*β* superfamily member, promotes thermogenic gene expression and subcutaneous WAT browning in mice, in part through p38 activation (158).

A number of other molecules have been reported to induce browning in a p38-dependent manner. Fibroblast growth factor 21 (FGF21) is induced in BAT by cold, in liver by nutrients and hormones, and in muscle by exercise (121) and acts as an inducer of WAT browning (159–162). FGF21 contributes to browning in three ways: by regulating the SNS through its action in the brain, by enhancing adrenergic stimulation of UCP1 (163), or by acting directly on white adipocytes to activate PGC1*α* (164). Another browning factor is the lipid sensor GPR120, which promotes browning in mice and adipocytes through p38-mediated FGF21 release (165). UCP1 and PGC1α are also induced by retinoic acid treatment in mice (166), apparently through p38 (167). The gut-restricted FXR agonist fexaramine (Fex) protects against diet-induced weight gain by promoting thermogenesis through the expression of UCP1 and PGC1*α* in WAT, also possibly *via* p38 (168). Browning is also promoted by the autocrine glycoprotein follistatin, which acts *via* p38 to increase the expression of beige adipocyte-specific markers and genes involved in thermogenesis, FA oxidation, and mitochondrial biogenesis in epididymal WAT and subcutaneous WAT (102). WAT browning in mice is stimulated by overexpression of zinc alpha 2 glycoprotein (ZAG) (169), and in 3T3-L1 white adipocytes ZAG increases the expression of brown fat markers and induces mitochondrial biogenesis *via* PKA and p38 signaling (170). The naturally occurring compound cryptotanshinone promotes brown-tissue and beige-cell markers by activating AMPK, p38, and Smad1/5 (58), whereas sinapic acid achieves the same effect *via* AMPK, p38, and PGC1*α* (171). Treatment with the non-steroidal anti-inflammatory drug ketoprofen induces browning in 3T3-L1 cells and inguinal WAT, increasing mitochondrial biogenesis through an intricate signaling pathway involving p38, mTORC1, and COX activation (172).

Until now, p38*α* was considered the family member implicated in browning, inducing UCP1 expression (13, 92). This assumption based largely on data from cell culture studies using p38*α* inhibitors, which blunt stimulation of the thermogenic program. However, non-specific effects of these inhibitors cannot be excluded, and several studies have shown that these inhibitors reduce p38 phosphorylation, revealing inhibition of upstream kinases, which in some cases might result in hyperactivation of other p38 isoforms (86, 102, 130, 132, 133, 151, 170). Despite these concerns about inhibitor specificity, studies with p38*α* genetic mutant mice were not reported until recently. Two parallel studies, one from our group, recently showed that ablation of p38*α* in mouse adipose tissue surprisingly activates the thermogenic program (10, 66). Mice with adipocyte p38*α* deficiency are lean, have improved metabolism, and are resistant to diet-induced obesity due to increased BAT thermogenesis and browning in inguinal WAT associated with enhanced CREB transcription activity (10, 66). However, we showed that in epididymal adipose tissue adipocyte p38*α* deficiency results in reduced browning (12). These studies show that the mechanisms controlling the thermogenic program, thought to be similar in brown and beige adipocytes, may differ between each fat depot. Distinct p38 family members may be implicated in these processes; thus, while p38*α* would block thermogenesis in BAT by inhibiting p38δ, in inguinal WAT it might abolish UCP1 expression by inhibiting p38*γ*, and in epidydimal WAT it could induce browning (10, 12).

## Role of p38 in Adipose Tissue Secretion

Adipose tissue is not only an energy reservoir, but also a secretory organ. The endocrine function of adipose tissue was discovered in the 1994 with the description of leptin (173). Since then, research has identified several other bioactive molecules secreted by adipocytes, called adipokines. Adipokines are released to the circulation and modulate metabolic processes in the body through actions on metabolic organs such as the liver and brain (174, 175). Adipokines are implicated in metabolic disorders such as obesity and are important targets in the treatment of obesity-related diseases. Adipokines with well-known regulatory functions include leptin, adiponectin, tumor necrosis factor α (TNFα), and interleukin-6 (IL6) (173, 176, 177). Adipokine secretion was initially thought to be limited to WAT, but lately it has become clear that BAT is also a source of adipokines with specific functions and produced in a distinct repertoire from that of WAT (74, 178).

The secretion of many of these adipokines likely involves SAPKs, which trigger the inflammatory response in many cells and control the secretion of pro-inflammatory mediators such as TNFα or IL6 (177, 179). Activation of adipocyte JNK1 is partially responsible for the increased levels of IL6 in obesity (8), and palmitate- or insulin resistance-stimulated JNK is also critical for adipocyte production of the chemokine MCP1. Chemical inhibition of JNK activity suppresses MCP1 release (180, 181). JNK also mediates the secretion of adipokines with non-inflammatory properties. Our group recently demonstrated that adipocyte JNK1 controls adiponectin levels in mice (182). Testosterone activates JNK in adipose tissue, and ablation of adipose JNK1 results in increased serum adiponectin, whereas activation of JNK in male adipose tissue correlates with reduced adiponectin levels (182).

There are promising data suggesting that p38 might regulate the adipose tissue secretome. In 2002, Finck and Johnson showed that chemical inhibition of p38 almost completely blocks TNFα-induced leptin expression in mice (183). Interestingly however, TNFα might have an opposite role in the regulation of leptin secretion. In the absence of corticosteroid medication with dexamethasone, TNFα inhibits leptin secretion, but in the presence of glucocorticoid treatment TNFα has the opposite effect. In cultured omental adipose tissue from dexamethasone-treated patients, the synergistic effect of TNFα on leptin secretion is blocked by p38 chemical inhibition, indicating that the effect of p38 on leptin secretion might be mediated by glucocorticoid receptor activation (184). More recently, Uchiyama et al. found, using a p38 chemical inhibitor in cultured adipocytes, that alamandin-decreased leptin expression was also mediated by p38 (185). Moreover, leptin expression is increased in *in vitro* differentiated p38*α*-deficient adipocytes (10). These studies thus suggest that p38 could be involved in leptin secretion by WAT; however, given the lack of specificity of the inhibitors used in these studies and their potential p38-independent effects, additional studies with genetic mouse models will be needed to fully define the role of p38 in the regulation of leptin secretion. p38 has also been suggested to regulate the secretion of adiponectin in obese human adipose tissue (186).

**Figure 5:**
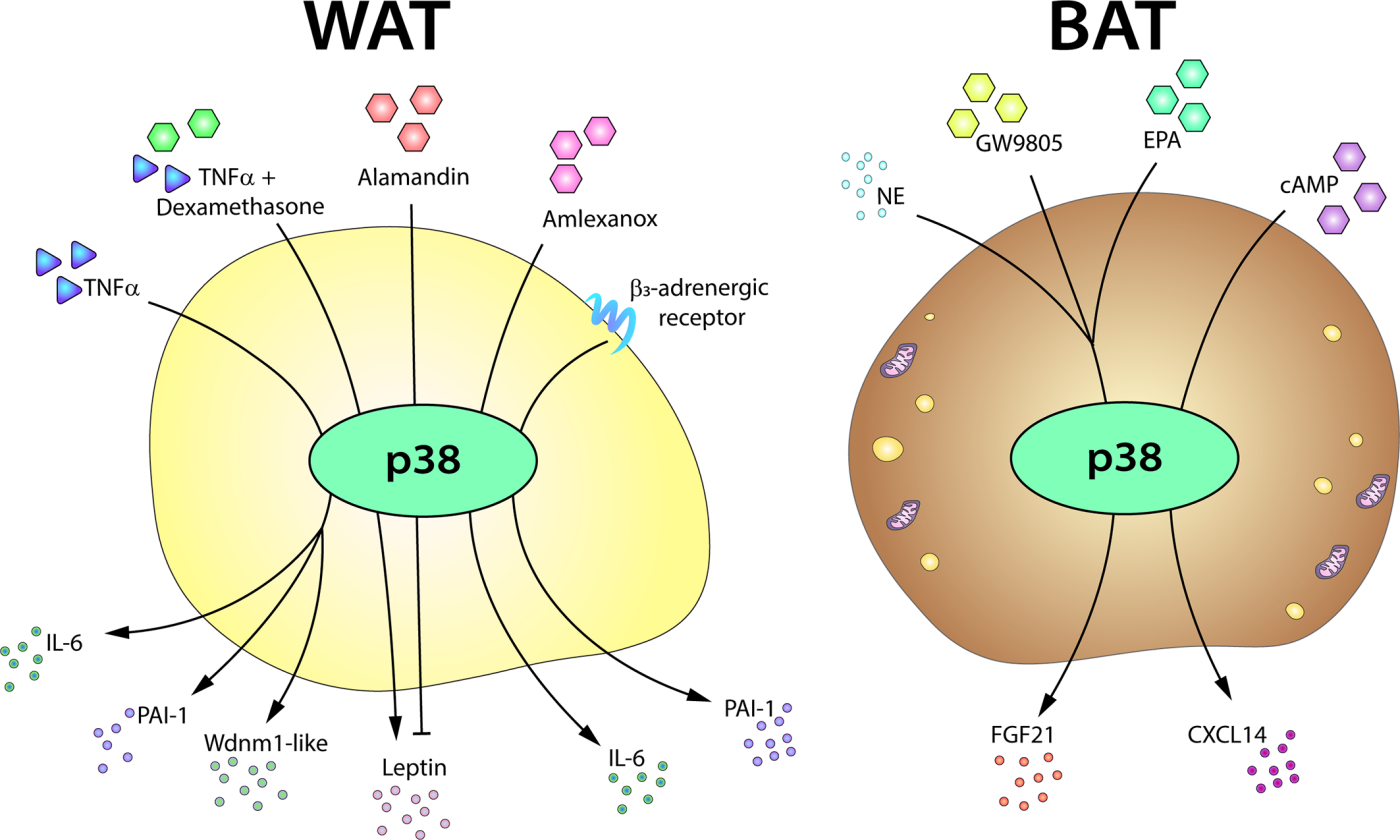
The p38-mediated adipose tissue secretome. The p38 pathway controls the expression or secretion of distinct adipokines in WAT and BAT. In white adipocytes, p38 activation is required for the production of IL6, PAI-1, and Wdnm1-like after TNFα stimulation. p38 signaling also controls leptin secretion in response to TNFα, playing a positive role in the presence of dexamethasone, but an inhibitory role in response to alamandin. p38 is also involved in the secretion of IL6 in response to amlexanox and in the induction of PAI-1 after *β*3AR stimulation. In brown adipocytes, p38 controls the secretion of FGF21 induced by NE, GW9805, or EPA and directs the secretion of CXCL14 after cAMP stimulation. cAMP, cyclic AMP; EPA, eicosapentanoic acid; FGF21, fibroblast growth factor 21; IL6, interleukin-6; NE, norepinephrine; PAI-1, plasminogen activator inhibitor-1; TNFα, tumor necrosis factor α.

There are also recent studies implicating the p38 pathway in the secretion of adipokines by brown adipocytes (187). The metabolic regulator FGF21 is secreted by BAT after thermogenic stimulation. Brown adipocyte expression of FGF21 requires p38 activation, and p38*α*/*β* inhibition impairs FGF21 transcriptional induction upon exposure to norepinephrine, the free-fatty acid receptor selective agonist GW9508, or eicosapentanoic acid (14, 165). Moreover, transfection of a brown adipocyte cell line with a constitutively active form of the p38 upstream kinase MKK6 increases FGF21 expression, while a dominant-negative form abolishes its induction by cAMP and PKA (14). Also during BAT thermogenesis, the cAMP-dependent chemokine CXCL14 recruits and polarizes macrophages to the M2 phenotype (188), and induction of *Cxcl14* mRNA in brown adipocytes is blunted by chemical inhibition of p38*α*/*β* (188). Nevertheless, further experiments are needed in mice lacking p38*α*/*β* or their upstream kinases to confirm the role of p38 signaling in CXCL14 secretion (**Figure 5**).

## Role of p38 in Adipose Tissue Inflammation

The p38 pathway was originally described as a master regulator of pro-inflammatory cytokine secretion in myeloid cells. In macrophages, different p38 family members are involved in the production of pro-inflammatory mediators (188, 189) and promote monocytes recruitment exacerbating the adipocyte pro-inflammatory response (189). Adipocytes are a major source of TNFα and IL6, and p38 inhibition partially blocks adipocyte TNFα-induced IL6 secretion, suggesting that the regulatory actions of p38s observed in macrophages might also operate in adipose tissue (184). Moreover, siRNA or chemical p38 inhibition impairs IL6 secretion by amlexanox-treated 3T3-L1 adipocytes, and this inhibitory effect is also observed in WAT from mice treated with p38 inhibitors (190). In this study, inhibition of IKK*ε*/TBK1 by amlexanox led to an increase in cAMP levels that triggered p38 activation in adipocytes and subcutaneous WAT (190). This is interesting because the cAMP–p38 pathway is crucial for the thermogenic process in BAT (13). Given that IKK*ε*/TBK1 inhibition does not increase IL6 expression in BAT, this mechanism might be specific to subcutaneous WAT depots, which are enriched in browning-susceptible beige adipocytes. IL6 has been shown to be required for the induction of browning in subcutaneous depots after cold exposure (191).

TNFα is chronically elevated in adipose tissue from obese mice and humans and is a main activator of the p38 pathway in several cell types (5). These abnormal levels of TNFα lead to the activation of stress signaling cascades that stimulate lipolysis in adipocytes impairing lipid handling capacity (192). The involvement of JNK in TNF-α stimulation of lipolysis in human adipocytes have been demonstrated (193), however a possible role of p38 family members in TNF-induced lipolysis need to be clarified. TNFα has been proposed to induce plasminogen activator inhibitor-1 (PAI-1) in adipocytes, an adipokine that contributes to the cardiovascular and metabolic complications associated with obesity (194). p38 inhibition impairs TNFα-induced PAI-1 expression in 3T3-L1 adipocytes, suggesting that this PAI-1 expression is p38 dependent (195). Similar results were found in a later study, in which p38 chemical inhibition partially decreased PAI-1 expression induced by β3AR activation in 3T3-L1 adipocytes (196). Nevertheless, chemical inhibition of p38 did not have the same effect in epididymal WAT from mice treated with a *β*3AR agonist (196). p38 inhibition also blocks TNFα induction of Wdnm1-like adipokine in 3T3-L1 adipocytes (197). These data are consistent with p38 mediation of TNFα-regulated adipokine expression (**Figure 5**). However, additional studies with animal models will be needed to confirm the role of p38 in the secretion of these adipokines.

Pro-inflammatory signals are important players of the BAT and beige adipocytes thermogenesis. Many inflammatory cells and pro-inflammatory cytokines secreted by both immune cells and adipocytes can negatively affect the thermogenic activation and browning of WAT (198). As it has been commented above, p38 controls the expression of many pro-inflammatory cytokines by macrophages (199) and also controls the secretion of the M2 chemokine CXCL14 (188). In addition, upon cold exposure, mice lacking adipose p38*α* have an increased mRNA expression of M2-related genes, a reduction in mRNA levels of pro-inflammatory cytokines, and an elevated macrophage infiltration in inguinal WAT (66). These results are in agreement with the increased browning in this mouse model, given that pro-inflammatory M1 macrophages infiltration in BAT impairs its ability to respond to thermogenic stimuli (200–202), while M2 macrophages are proposed to be directly involved in promoting BAT thermogenesis (203).

## Future Perspectives

Adipose tissue is an important organ in metabolic regulation. Much more than a simple fuel reservoir, adipose tissue has endocrine functions and is able to massively expand or retract according to the nutrient supply. This unique, almost unlimited capacity of adipose tissue is critical for maintaining homeostasis, and its disturbance is directly linked to obesity and related disorders and comorbidities. Increasing knowledge about adipose tissue biology has led to a better understanding of its role in health and disease. p38 stress kinases are one of a major regulators of cell adaptation to external and internal changes and play important roles in adipose tissue. In recent years, significant advances have defined the roles of p38 kinases in different adipocyte cell types. However, the use of pharmacological inhibitors in most studies and the limited data from genetically modified animals show the need for caution in interpreting these findings while further research is undertaken. The use of chemical inhibitors has important limitations because of their non-specificity and side effects. Additionally, inhibitors with selective activity against p38*α* and p38*β* might affect p38*γ* and p38*δ* activation due to the negative feedback exerted by p38*α* on the upstream activators of the pathway, MKK3/MKK6. Other hurdles to progress are the lack of antibodies recognizing the phosphorylated form of each family member and the small number of substrates identified for the less studied family members. Research in the coming years, using appropriate genetically modified animal models and evaluating the phosphorylation of specific substrates of each family member, is expected to better define the role of the all p38s in adipose tissue. For example, research in adipogenesis would need to assess the role of each p38 in the differentiation of adipose tissue in steady-state and in obesity. More importantly, research is needed to identify the main p38 family member involved in human adipose tissue browning and BAT activation in order to define the potential of this pathway as a target for obesity treatment.

BAT activation and browning have emerged as therapeutic targets for the treatment of obesity and its associated diseases. We recently demonstrated that inhibition of p38*α* leads to the hyperactivation of p38*δ* and that active p38*δ* increases thermogenesis. This finding has important clinical implications because p38*α* inhibition or p38*δ* activation might be feasible therapeutic approaches to the treatment of obesity-related disorders. What is required now is the design of selective inhibitors or activators targeting the appropriate p38 family member. These specific molecules might allow prevent the progression of obesity and treat clinical obese patients.

Another important goal is the identification of p38-dependent cytokines, adipokines or batokines susceptible to modulation by manipulation of the p38 pathway. The existing evidence of an association between some adipokines and batokines and the p38 pathway will need to be confirmed using appropriate genetic tools.

From the evidence explored in this review, it is clear that targeting the p38 pathway in adipose tissue can provide a firm and timely understanding of obesity and its associated pathologies in the era of the global obesity pandemic.

## Author Contributions

Each author contributed to the literature search and wrote a section of the manuscript. IN drew the figures, which were revised by all authors. All authors contributed to the article and approved the submitted version.

## Funding

ML was supported by a Spanish grant MINECO-FEDER-SAF2015-74112-JIN and Fundación AECC: INVES20026LEIV. MP was a fellow of the Spanish State programme for the Promotion of Talent and its Employment (PRE2018-083631). IN was funded by the EFSD/Lilly grants (2017 and 2019), the CNIC IPP FP7 Marie Curie Programme (PCOFUND-2012-600396), an EFSD Rising Star award (2019), and the JDC-2018-Incorporación (MIN/JDC1802). GS received funding from the following programs and organizations: European Union Seventh Framework Programme (FP7/2007-2013) under grant agreement n° ERC 260464, the EFSD/Lilly European Diabetes Research Programme, the BBVA Foundation Leonardo Grants program for Researchers and Cultural Creators (Investigadores-BBVA-2017) IN[17]_BBM_BAS_0066, MINECO-FEDER SAF2016-79126-R, and the Comunidad de Madrid (IMMUNOTHERCAN-CM S2010/BMD-2326 and B2017/BMD-3733). The CNIC is supported by the Instituto de Salud Carlos III (ISCIII), the Ministerio de Ciencia, Innovación y Universidades (MCNU), and the Pro CNIC Foundation and is a Severo Ochoa Center of Excellence (SEV-2015-0505).

## Conflict of Interest

The authors declare that the research was conducted in the absence of any commercial or financial relationships that could be construed as a potential conflict of interest.
